# Color and Relative Translucency of Three-Dimensionally Printed Restorative Resin Systems: Effects of Build Orientation, Thickness and Staining Conditions

**DOI:** 10.3390/polym18202524

**Published:** 2026-10-15

**Authors:** Hilal Gülgezen Aydın, Gülçin Cagay Sevencan

**Affiliations:** 1 Department of Prosthodontics, Faculty of Dentistry, Tekirdağ Namık Kemal University, 59030 Tekirdağ, Türkiye; 2 Department of Endodontics, Faculty of Dentistry, Tekirdağ Namık Kemal University, 59030 Tekirdağ, Türkiye

**Keywords:** spectrophotometry, colorimetry, composite resins, dental restoration, permanent, materials testing

## Abstract

This study assessed the effects of material system, build orientation, thickness, and immersion time on optical properties. A total of 224 discs (*n* = 7 per subgroup) were fabricated from Permanent Crown Resin (system F) and Saremco Print CROWNTEC (system S) at 0° and 90° orientations and 1- and 2-mm thicknesses. Specimens were immersed in distilled water, coffee, tea, or red wine and measured at baseline and days 1, 7, and 30. Baseline CIELAB coordinates and changes in color (ΔE_00_) and relative translucency parameter (ΔRTP_00_) were analyzed by three-way and medium-specific four-factor mixed analyses of variance, respectively (α = 0.05). Material system dominated baseline variation (η_p_^2^ = 0.70–0.94); system S was lighter with lower a^*^ and b^*^ values. Time affected ΔE_00_ in all media and ΔRTP_00_ in staining media (*p* < 0.001). All between-specimen factors affected both outcomes in staining media, with medium-specific interactions. Descriptively, red wine produced the greatest changes; all red-wine groups exceeded the color acceptability threshold at day 30. System F at 90° and 1 mm showed the largest changes (ΔE_00_ = 41.46 ± 9.48; ΔRTP_00_ = 5.57 ± 0.85). No 2-mm group exceeded the translucency acceptability threshold. Optical behavior depended on configuration and exposure. System S showed greater color stability, mainly at 0°, whereas 90° orientation and reduced thickness generally increased optical change. Material, printing and post-polymerization effects could not be separated.

## 1. Introduction

The esthetic integration of tooth-colored restorations depends on matching the natural dentition in color and translucency and maintaining these properties over time. Color assessment is typically done using the Commission Internationale de l’Éclairage (CIE) L^*^a^*^b^*^ color space, in which L^*^ indicates lightness, and a^*^ and b^*^ represent red–green and yellow–blue chromatic axes, respectively [1]. The CIEDE2000 color-difference formula (ΔE_00_) aligns better with visual perception and allows interpretation of color differences using perceptibility and acceptability thresholds [2]. Advances in digital dentistry have extended additive manufacturing from interim to definitive resin-based restorations, but the reproducibility and long-term stability of their optical properties remain clinical concerns [3,4].

The color stability of resin-based materials is influenced by intrinsic factors like composition and polymerization degree, and extrinsic factors such as exposure to chromogenic agents. Inorganic filler content, particle size and refractive-index differences between the filler and the polymer matrix influence light scattering and can thereby affect the color and translucency of resin composites [5]. Beverages like coffee, tea, and especially red wine cause clinically perceptible color changes. Studies suggest that three-dimensionally (3D) printed resin-based composites may exhibit greater susceptibility to beverage-induced color changes than milled materials, thereby raising concerns about their long-term esthetic performance [6,7,8,9]. Immersion in water or staining media may alter both color and translucency; therefore, evaluating color change alone may not fully characterize the optical stability of resin-based composites. Established translucency perceptibility and acceptability thresholds help interpret these changes clinically [10].

In addition to the material system, geometric and manufacturing parameters may affect optical behavior. Restoration thickness determines the optical path length and transmitted light, while build orientation alters the relationship between printed layers, interlayer interfaces, and incident light [4,11,12]. Previous studies have reported that thickness and build orientation can affect the color and translucency of 3D-printed resin-based composites, but results vary by material and manufacturing protocol. Most evidence focuses on interim materials or examines factors separately. Since commercial definitive restorative resin-based composites use manufacturer-specific printing and post-polymerization protocols, product comparisons reflect entire material-processing systems rather than material composition alone. The independent and combined effects of material system, build orientation, and thickness on baseline color coordinates and time-dependent optical changes remain poorly understood. This study extends existing knowledge by jointly evaluating color and relative translucency changes and examining interactions among material system, build orientation, thickness and immersion time within each storage medium. This factorial approach clarifies how specific combinations of these factors influence optical stability in definitive 3D-printed resin-based composites.

Optical factors are crucial in restoring endodontically treated teeth, balancing esthetics and tooth structure preservation [13]. Since 3D-printed resin-based composites have been experimentally used as one-piece endodontic crowns, this study evaluated how material system, thickness, and build orientation affect optical stability, offering evidence for their use in post-endodontic rehabilitation [14].

This in vitro study aimed to evaluate baseline CIE L^*^, a^*^, and b^*^ color coordinates of two commercial 3D-printed resin-based composites intended for definitive restorations, fabricated at two thicknesses and two build orientations using their respective fabrication workflows. Within each storage medium, it also assessed color change (ΔE_00_) and changes in the relative translucency parameter (ΔRTP_00_) over 30 days. The first null hypothesis stated that baseline CIE L^*^, a^*^, and b^*^ values would not differ by material system, build orientation, thickness, or their interactions. The second hypothesized that, within each storage medium, ΔE_00_ would not vary by material system, build orientation, thickness, immersion time, or their interactions. The third posited that ΔRTP_00_ would similarly show no differences based on these factors or their interactions.

## 2. Materials and Methods

No human participants, animals, or biological tissues were involved; thus, ethics approval was not required. Two A2-shade resin-based composites were studied: Permanent Crown Resin (Formlabs Inc., Somerville, MA, USA) and Saremco Print CROWNTEC (Saremco Dental AG, Rebstein, Switzerland), designated systems F and S, respectively. According to manufacturer-reported compositions, both materials contain an organic resin matrix and inorganic fillers. Each material system included the composite material and its manufacturer-specific printing, cleaning and post-polymerization processes. Since each material was processed only with its corresponding workflow, material composition and printing technology could not be separated experimentally; comparisons refer to the entire material systems rather than material composition alone. Compositions and fabrication workflows are summarized in Table 1.

The experiment involved 2 material systems, 2 build orientations (0° and 90°), 2 specimen thicknesses (1 and 2 mm), and 4 storage media (distilled water, coffee, tea, and red wine). Immersion time was measured repeatedly at baseline (t_0_) and after 1 (t_1_), 7 (t_7_), and 30 days (t_30_). Each combination of system, orientation, thickness, and medium included 7 specimens, totaling 224 specimens (2 × 2 × 2 × 4 × 7).

An a priori sample size calculation was conducted using sample-size software (G*Power, version 3.1.9.7; Heinrich Heine University Düsseldorf, Düsseldorf, Germany) with a 0.05 type I error rate, 0.95 power, and an effect size of 1.01 based on prior orientation-related color differences [4]. This required at least 28 specimens per material system, or 7 for each of the 4 orientation–thickness groups. For the longitudinal staining experiment, this allocation was applied separately across 4 storage media, resulting in 112 specimens per system. Specimens within each system–orientation–thickness group were assigned unique identification numbers and randomly distributed equally among the four storage media.

Disc-shaped specimens (10 mm in diameter; nominal thickness, 1 or 2 mm) were designed with dental design software (DentalCAD, version 3.2 Elefsina, 2023; exocad GmbH, Darmstadt, Germany) and exported as standard tessellation language files. Build orientation was defined as the angle between the broad surface of the disc and the build platform: the broad surface was parallel to the platform at 0° and perpendicular to it at 90°. These standardized orientations were selected as controlled boundary conditions for evaluating the orientation effect. Layer thickness was set to 50 µm for both systems. Both printers used vat photopolymerization to fabricate the specimens and did not employ material-extrusion nozzles.

System S specimens were additively manufactured with a digital light-processing printer (Asiga MAX UV; Asiga, Alexandria, NSW, Australia). After separation from the build platform with a putty knife, excess uncured material was removed with 96% alcohol wipes (Wipex 96% Isopropyl Alcohol Wipes; Dutch Harbor Brands, Boca Raton, FL, USA). The specimens were then brushed with 99% isopropyl alcohol until no visible residues of uncured material remained and dried with an air syringe. Post-polymerization was performed under nitrogen in a light-polymerization unit (Otoflash G171; NK-Optik GmbH, Baierbrunn, Germany) for two cycles of 2000 flashes, with the specimens rotated after the first cycle.

System F specimens were additively manufactured with a stereolithography printer (Form 3; Formlabs Inc., Somerville, MA, USA). The specimens were washed for 3 min in 99% isopropyl alcohol in a washing unit (Form Wash; Formlabs Inc., Somerville, MA, USA), dried with an air syringe, and post-polymerized in a polymerization unit (Form Cure; Formlabs Inc., Somerville, MA, USA) for 20 min at 60 °C. For both systems, support structures were removed with a cut-off wheel. All specimens were sequentially polished under water with 400-, 600-, and 1200-grit silicon carbide abrasive papers for 15 s per grit to standardize the surfaces. The sequence was performed once without returning to a previous grit. Final diameter and thickness were verified with a digital caliper (Absolute Digimatic; Mitutoyo Corp., Kawasaki, Japan), and the specimens were stored in the dark until baseline measurements.

Baseline optical measurements were obtained immediately before immersion. Distilled water served as the control medium. The coffee medium was prepared by brewing 3 g of granulated coffee (Nescafé Gold; Nestlé S.A., Vevey, Switzerland) in 300 mL of boiling distilled water for 10 min and filtering the solution. The tea medium was prepared by steeping one tea bag of black tea (Lipton Yellow Label; Unilever Korea Co., Seoul, Republic of Korea) in 500 mL of boiling distilled water for 15 min. Commercial red wine (Doluca Red; Doluca Wine Co., Istanbul, Türkiye) was used without dilution.

The specimens were stored in their assigned media in the dark at 37 ± 2 °C for 30 days, and all media were renewed every 2 days. The same specimens were evaluated at t_1_, t_7_, and t_30_. At each interval, specimens were removed from the medium, rinsed with distilled water for 5 min, blotted dry with tissue paper, and measured. After the t_1_ and t_7_ measurements, they were returned to their corresponding freshly prepared media.

CIE L^*^, a^*^, and b^*^ coordinates were measured with a digital spectrophotometer (VITA Easyshade V; VITA Zahnfabrik, Bad Säckingen, Germany) at t_0_, t_1_, t_7_, and t_30_. A previous study estimated that 24 h of continuous immersion in coffee corresponded to approximately 1 month of coffee consumption, assuming 15 min of contact per cup and 3.2 cups per day [15]. Measurements were performed under D65 illumination with the CIE 2° standard observer against neutral gray, white, and black backgrounds [16]. The background coordinates were as follows: gray, L^*^ = 20.0, a^*^ = 0.2, and b^*^ = 3.2; white, L^*^ = 53.2, a^*^ = 3.9, and b^*^ = 13.5; and black, L^*^ = 9.2, a^*^ = −0.3, and b^*^ = 1.5.

The measuring tip, 5 mm in diameter, was positioned at the center of each 10 mm diameter disc. Measurements were obtained from the central region rather than the entire disc surface. The spectrophotometer was calibrated according to the manufacturer’s instructions before the measurement series and recalibrated between experimental groups. Three consecutive readings were obtained for every specimen on each background, and their mean was used in the analyses. All measurements were performed by the same examiner using the same standardized protocol.

Color differences between baseline and each immersion interval were calculated from the CIE L^*^, a^*^, and b^*^ coordinates measured against the neutral gray background using the CIEDE2000 color-difference formula (ΔE_00_). ΔE_00_ values were calculated for t_1_–t_0_, t_7_–t_0_, and t_30_–t_0_. The CIEDE2000 equation was applied as follows [17,18]:

(1) $${∆E}_{00}=\sqrt{{\left(\frac{∆L^{\prime }}{k_{L}\;S_{L}}\right)}^{2}+\;{\left(\frac{∆C^{\prime }}{k_{C}\;S_{C}}\right)}^{2}+\;{\left(\frac{∆H^{\prime }}{k_{H}\;S_{H}}\right)}^{2}+R_{T\;}\left(\frac{∆C^{\prime }}{k_{C}\;S_{C}}\right)\;\left(\frac{∆H^{\prime }}{k_{H}\;S_{H}}\right)}$$

In the CIEDE2000 formula, Δ*L′*, Δ*C′* and Δ*H′* represent the lightness, chroma and hue difference terms, respectively. The prime notation identifies quantities defined within the CIEDE2000 formulation; lightness remains unchanged (L′ = L*). *S_L_*, *S_C_* and *S_H_* are the corresponding perceptual weighting functions. *R_T_* accounts for the interaction between chroma and hue differences in the blue region. The parametric factors *k_L_*, *k_C_* and *k_H_* were set to 1 for both ΔE_00_ and RTP_00_ calculations. Color differences were interpreted using the 50:50% perceptibility (PT_00_ = 0.81) and acceptability (AT_00_ = 1.81) thresholds [17,19].

The relative translucency parameter (RTP_00_) was defined as the CIEDE2000 color difference between measurements over black (B) and white (W) backgrounds and calculated as follows [1,10]:

(2) $${RTP}_{00}\;=\;\sqrt{{\left(\frac{{L^{\prime }}_{B}-{L^{\prime }}_{W}}{k_{L}S_{L}}\right)}^{2}\;+\;{\left(\frac{{C^{\prime }}_{B}-{C^{\prime }}_{W}}{k_{C}S_{C}}\right)}^{2}+\;{\left(\frac{{H^{\prime }}_{B}-{H^{\prime }}_{W}}{k_{H}S_{H}}\right)}^{2}+\;R_{T\;}\left(\frac{{C^{\prime }}_{B}-{C^{\prime }}_{W}}{k_{C}S_{C}}\right)\;\left(\frac{{H^{\prime }}_{B}-{H^{\prime }}_{W}}{k_{H}S_{H}}\right)}\;$$

Changes in the relative translucency parameter between time points (ΔRTP_00_) were calculated as follows [1]:

(3) ΔRTP_00_ = RTP_002_–RTP_001_

Translucency differences were interpreted using published 50:50% perceptibility (TPT_00_ = 0.62) and acceptability (TAT_00_ = 2.62) thresholds [10]. In the reference study, these thresholds corresponded to translucency differences detected by 50% of observers and judged acceptable by 50% of observers, respectively.

Analyses were conducted using statistical software (SPSS Statistics, version 29.0; IBM Corp., Armonk, NY, USA). Descriptive statistics are presented as means and standard deviations. Normality was assessed with Shapiro–Wilk tests and Q–Q plots, while homogeneity of variances was evaluated using Levene’s test.

Baseline L^*^, a^*^, and b^*^ coordinates were analyzed using three separate three-way factorial analyses of variance (ANOVAs), with material system, build orientation, and thickness as between-specimen factors. Each model included all two-way and the three-way interactions. Storage medium was excluded since measurements were taken before immersion.

Separate four-factor mixed ANOVAs were conducted for ΔE_00_ and ΔRTP_00_ within each storage medium. Material system, build orientation, and thickness were between-subject factors, while time (t_1_–t_0_, t_7_–t_0_, and t_30_–t_0_) was the within-subject factor. All interactions among the four factors were included. Each medium was treated as a distinct experimental environment, so no inferential comparisons were made between storage media.

Sphericity, defined as equality of the variances of the differences between all pairs of repeated measurements, was assessed using Mauchly’s test. When this assumption was violated, Greenhouse–Geisser corrections were applied to the degrees of freedom and the corresponding adjusted *p*-values were used. Significant main effects or interactions were analyzed with Bonferroni-adjusted estimated marginal means. Effect sizes are reported as partial eta squared (partial η^2^). All tests were two-sided with a significance level of α = 0.05.

## 3. Results

Baseline CIELAB coordinates and three-way analysis of variance results are summarized in Table 2. Across matched orientation–thickness combinations, system S showed higher L^*^ and lower a^*^ and b^*^ values than system F. For L^*^, all main effects and two-factor interactions were significant (all *p* < 0.001), whereas the three-factor interaction was not (*p* = 0.195). For a^*^, all main effects, material system × build orientation, build orientation × thickness, and the three-factor interaction were significant (all *p* < 0.001); material system × thickness was not (*p* = 0.083). For b^*^, build orientation was significant at *p* = 0.001, all other significant effects had *p* < 0.001, and material system × build orientation was not significant (*p* = 0.184). Material system explained the largest proportion of variance in L^*^ (partial η_p_^2^ = 0.82), a^*^ (partial η_p_^2^ = 0.94), and b^*^ (partial η_p_^2^ = 0.70).

Descriptive ΔE_00_ values are presented in Table 3, and the four-factor mixed analysis of variance results for each storage medium are shown in Table 4. Time significantly affected ΔE_00_ in every medium (all *p* < 0.001; partial η_p_^2^ = 0.52–0.97). In distilled water, build orientation was the only significant between-specimen main effect (*p* < 0.001). The material system × specimen thickness, time × material system, and time × build orientation interactions were also significant (*p* = 0.046, *p* < 0.001, and *p* = 0.006, respectively). In coffee, tea, and red wine, the main effects of material system, specimen thickness, and build orientation were all significant (all *p* < 0.001). Build orientation was the strongest between-specimen factor in coffee (partial η_p_^2^ = 0.93) and tea (partial η_p_^2^ = 0.89), whereas material system was the strongest between-specimen factor in red wine (partial η_p_^2^ = 0.84). Significant time-dependent interactions indicated that the magnitude of change varied across material systems, thicknesses, and build orientations.

**Figure 1:**
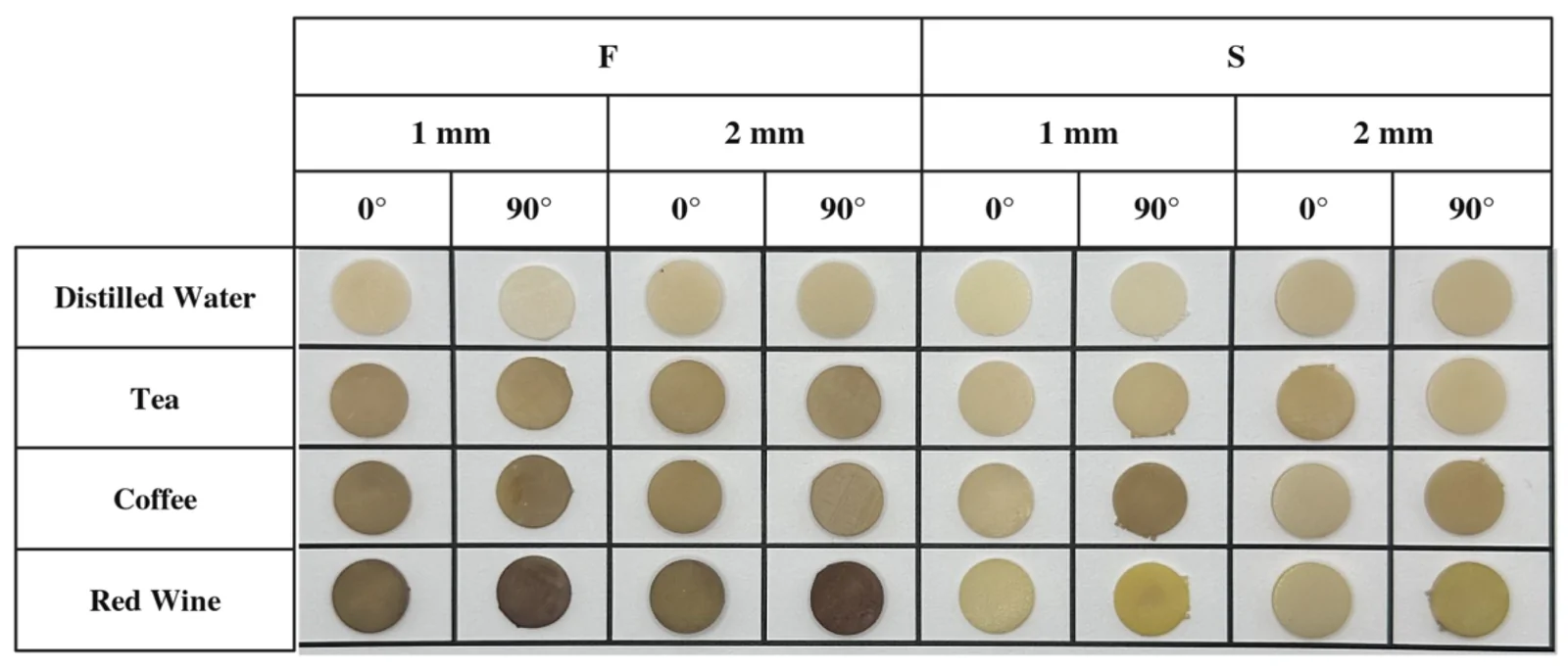
Representative specimens after 30 days of immersion in distilled water, tea, coffee and red wine, arranged by material system, specimen thickness and build orientation. F: Formlabs Permanent Crown; S: Saremco print CROWNTEC.

At day 30, the 50:50% acceptability threshold (AT_00_ = 1.81) was exceeded in distilled water by the F/90°/1-mm, F/90°/2-mm, and S/90°/1-mm groups. In coffee, all F groups and both S groups printed at 90° exceeded AT_00_; in tea, all F groups and all S groups except S/0°/2 mm exceeded AT_00_. Every red-wine group exceeded AT_00_. The largest color change was observed for F/90°/1 mm in red wine at day 30 (ΔE_00_ = 41.46 ±9.48). Representative specimens after immersion in the different media are shown in Figure 1.

Descriptive ΔRTP_00_ values and the corresponding mixed analysis of variance results are presented in Table 5 and Table 6, respectively. Time had no significant main effect in distilled water (*p* = 0.274), although specimen thickness was significant (*p* < 0.001), and several time-dependent interactions involving build orientation were detected (Table 6). Time significantly affected ΔRTP_00_ in coffee, tea, and red wine (all *p* < 0.001; partial η_p_^2^ = 0.60–0.79). Material system was significant in coffee (*p* = 0.011), tea (*p* = 0.040), and red wine (*p* < 0.001); specimen thickness and build orientation were significant in all three staining media (all *p* < 0.001). All time-related interactions, including the four-factor interaction, were significant in coffee (Table 6). In tea, the time × specimen thickness and time × material system × build orientation interactions were significant (*p* = 0.005 and *p* = 0.001, respectively); in red wine, the only significant time interaction was time × specimen thickness (*p* < 0.001).

At day 30, the translucency acceptability threshold (TAT_00_ = 2.62) was exceeded by the F/90°/1-mm and S/90°/1-mm coffee groups, the S/90°/1-mm tea group, and the F/0°/1-mm, F/90°/1-mm, and S/90°/1-mm red-wine groups. No group stored in distilled water exceeded TAT_00_. The largest translucency change occurred for F/90°/1 mm in red wine at day 30 (ΔRTP_00_ = 5.57 ±0.85).

## 4. Discussion

The first null hypothesis was rejected, whereas the second and third were partially rejected. Baseline coordinates were affected by material system, build orientation, thickness, and several interactions. For ΔE_00_, time was significant in all media and all between-specimen factors in the staining media, whereas distilled water showed a more selective pattern. For ΔRTP_00_, time and all between-specimen factors were significant in the staining media; in distilled water, time was not significant, although thickness and selected interactions remained significant. Overall, optical behavior depended on the combination of factors and exposure conditions. Color and relative translucency changes showed different responses to the experimental factors. Among the between-specimen factors, build orientation showed the largest effect on color change in coffee and tea, whereas resin system showed the largest effect in red wine. Specimen thickness significantly affected relative translucency change in all media. Significant interactions indicated that the effects of individual factors depended on their combinations and, for time-related interactions, on immersion duration.

At baseline, the material system accounted for the largest variance in L^*^, a^*^, and b^*^ (partial η_p_^2^ = 0.70–0.94). System S was lighter with lower a^*^ and b^*^ values than system F across matched orientation-thickness combinations. However, significant interactions qualified these differences. For L^*^, all two-factor interactions were significant: increasing thickness reduced lightness in both systems, while orientation-related L^*^ reduction was pronounced in system F but limited and configuration-dependent in system S. The a^*^ coordinate generally increased at 90°, with a significant three-factor interaction showing this change depended on system and thickness. The b^*^ pattern was highly configuration-specific, evidenced by significant system × thickness, orientation × thickness, and three-factor interactions. Similar orientation- and thickness-dependent optical variations have been reported for additively manufactured restorative resin-based composites [4,11,20,21]. Thus, interpreting build orientation or thickness as isolated, uniform effects oversimplifies the findings.

Differences in baseline coordinates and longitudinal optical behavior may stem from formulation and fabrication variations. The color of resin-based materials depends on light absorption and scattering, influenced by the resin matrix, filler characteristics, pigment distribution, and refractive-index matching [5,22]. Both systems, as shown in Table 1, contain a Bis-EMA-based matrix and 30–50 wt% inorganic filler but differ in filler type and photoinitiator composition: system F uses TPO, while system S contains methyl benzoylformate and TPO. Variations in monomer composition and polymer conversion may contribute to optical differences [23,24]. The same applies to photoinitiator chemistry, filler size and distribution, and pigment stability or water/chromogen uptake [24,25,26,27,28]. Material viscosity affects layer formation and polymerization kinetics [29]. However, overlapping manufacturer-reported ranges (Table 1) suggest viscosity alone does not explain the findings. Sequential layer polymerization may also cause variations in conversion and residual monomer content, increasing staining susceptibility [30,31]. The compositional information in Table 1 was obtained from the manufacturers. As no independent chemical characterization of the materials was performed, the proposed links between composition and optical behavior should be considered possible explanations.

The fabrication workflows were not interchangeable: System F was produced by SLA, and system S by DLP, with differing cleaning and post-polymerization procedures. These variations may affect light exposure, polymer network development, curing uniformity, and surface morphology [9,31,32,33,34]. Thus, this design cannot isolate whether differences stem from material composition, printer technology, or post-processing. Consequently, the findings should be viewed as comparisons between two complete material systems and workflows, rather than isolated material effects or a direct SLA versus DLP comparison. Microporosities and incomplete polymerization at layer interfaces may increase the discoloration susceptibility of printed materials during immersion [30]. Filler particle size and distribution may influence translucency by altering light scattering, with the magnitude of this effect depending on filler content and refractive-index matching between the filler and the resin matrix [22].

Color change increased over time in all storage media, but its magnitude and temporal pattern differed across configurations. In distilled water, build orientation was the main factor, with 90° specimens showing greater changes and an increasing orientation-related difference over time. System S exhibited an early increase followed by stabilization, while system F continued changing through day 30. The system × thickness interaction indicated that increased thickness did not consistently reduce color change in water. In coffee and tea, build orientation remained dominant. System S showed greater color stability at 0°, but this advantage diminished at 90°, whereas system F experienced substantial changes at both orientations. At 90°, the layer boundaries intersect the measured surface. If pores or irregularities are present at these interfaces after polishing, they may provide sites for physical retention of beverage pigments, alongside possible chemical interactions with the resin matrix. This could contribute to the reduced color stability observed at this orientation. However, surface porosity and pigment penetration were not assessed, so this explanation remains hypothetical. Greater thickness was often associated with smaller measured color changes, although this pattern depended on the material system and build orientation. In red wine, the material system/workflow was the primary factor, with system F being especially susceptible, particularly at 90° and 1-mm thickness. These results demonstrate that long-term color stability cannot be predicted by material system, orientation, or thickness alone.

Each storage medium was modeled separately, so no inferential comparison between media was made; their apparent ranking should be viewed descriptively. Red wine caused the greatest color changes, consistent with its high staining potential reported previously [6,35,36]. Its low pH, alcohol content, and chromogens may promote resin-matrix softening and pigment penetration [28,37]. However, without a matched ethanol-free control, this study could not establish whether the same chromogens would produce comparable color changes in the absence of ethanol. Coffee and tea also caused progressive staining, but the strong orientation dependence suggests that beverage exposure alone did not determine the response. Adsorption and absorption of chromogens, along with their polarity and affinity for the polymer network, may explain system- and configuration-specific differences [28,38,39,40]. The release of residual photoinitiator components into water has been suggested as a possible explanation for color changes during water storage [24]. At day 30 in distilled water, mean ΔE_00_ values remained below the acceptability threshold in all 0° groups, whereas the 90° groups exceeded it except for the 2-mm S group. This orientation-related pattern is consistent with previous reports showing that build orientation can affect the color stability of 3D-printed resin materials [20]. In the 2-mm S group, the 0° mean was below the perceptibility threshold and the 90° mean was between the two thresholds. Layer orientation may influence water access at interfaces and thereby contribute to these color changes. However, these mechanisms were not directly assessed. Overall, staining susceptibility reflects an interaction among the chemical environment, resin-system workflow, and anisotropic structure from layer-by-layer fabrication [20,30,33].

Relative translucency changes were less extensive than color changes and followed a different pattern. Relative translucency is an instrumental expression of the change in a restoration’s measured appearance when the underlying background changes. Changes in relative translucency may be visually perceived and can influence the overall esthetic appearance of a restoration [10]. Although this property is generally more visually critical in anterior restorations, it may also contribute to the appearance of posterior restorations, particularly when they are thin or when the underlying substrate influences the final appearance. In the present study, ΔRTP_00_ provides an instrumental estimate of changes in relative translucency and does not represent a direct observer-based assessment. In distilled water, ΔRTP_00_ did not increase uniformly over time; thickness was the main factor, and no group exceeded the acceptability threshold. In staining media, ΔRTP_00_ increased over time, with thickness as the most consistent determinant. System–orientation effects varied by medium: coffee and tea showed configuration-dependent responses, with system behavior changing by orientation, while in red wine, thickness primarily governed changes, with system F generally altering more than system S and reduced thickness having a greater effect at 90°. No 2-mm configuration exceeded the translucency acceptability threshold across all media and times, suggesting greater thickness buffers light transmission changes but does not uniformly protect color. The divergence between ΔE_00_ and ΔRTP_00_ indicates that clinically relevant color shifts do not necessarily correspond to similar translucency changes. Chromogen uptake may alter color before absorption and scattering affect light transmission enough to cross the threshold. With deeper penetration, pigments may enter interlayer regions, affecting both outcomes [5,20,30,41]. The greater ΔE_00_ values observed in thinner specimens do not necessarily indicate greater surface susceptibility to staining. In translucent materials, measured color depends on specimen thickness and the combined optical contribution of the material and background [11]. These effects may influence how immersion-induced optical changes are expressed in the measured color difference. Because surface alterations and the depth of chromogen penetration were not assessed, the mechanism underlying the thickness-related differences could not be established.

The orientation effect likely stems from the anisotropic, multilayered structure of additively manufactured resins. At 0°, the layer planes align parallel to the measured surface, causing the incident beam to traverse stacked layers; at 90°, the planes are perpendicular, making the optical path more parallel to the layers and their boundaries. This shift may affect reflection, scattering and transmission [4,11,41]. Separately, build orientation may influence staining behavior [20]. Orientation-dependent curing and surface topography may also play roles [20,31,33]. Some studies report greater ΔE_00_ values or significant color differences in 90–oriented specimens, while others find no orientation effect [4,20,21,33]. Thickness-dependent changes in translucency and optical properties within the same material system have also been noted [42]. Smaller longitudinal changes in thicker and 0° configurations highlight the importance of adequate restoration thickness and layer orientation for esthetic stability. However, the present study shows that neither 0° orientation nor 2-mm thickness consistently protects across all systems, media, and outcomes. Overall, material system, specimen thickness, and build orientation collectively determine the optical stability of 3D-printed restorative resin-based composites.

Clinical interpretation of build orientation requires caution, as it also impacts printing time, support placement, accuracy, mechanical performance, and material use [43,44]. A curved, multisurface restoration cannot be globally aligned to the 0° or 90° orientations of a flat disc; its layer–surface relationship varies locally, and supports are typically placed on nonfunctional or less visible surfaces. Thus, the tested orientations represent boundary conditions illustrating optical anisotropy rather than universal printing guidelines. When anatomy, support design, and mechanical needs allow, maintaining adequate thickness and avoiding unfavorable local layer alignment may reduce long-term optical changes, especially in highly visible areas with limited restorative thickness or in patients frequently exposed to staining beverages. These findings may guide esthetic rehabilitation of endodontically treated teeth, emphasizing the importance of preserving residual tooth structure [13]. Greater optical changes in thinner specimens are relevant for printed resin-based composites in conservative restorations. However, the results do not justify additional tooth reduction, and their clinical significance needs evaluation in restorations on tooth substrates.

This in vitro study has several limitations. Continuous immersion does not mimic intermittent beverage exposure or saliva’s protective and cleansing effects. The design excluded thermal cycling, toothbrushing, mechanical wear, ultraviolet exposure, and cementation. Thus, the 30-day results reflect accelerated staining rather than direct clinical service time. Standardized polished discs improved measurement control but did not replicate restoration anatomy, variable thickness, curved surfaces, or clinical support locations. Only two manufacturer-specific systems, two boundary orientations, and thicknesses were tested. Additionally, system formulation and fabrication workflow were confounded, and storage media were not compared within a single inferential model. Surface roughness, surface porosity, filler particle-size distributions, water sorption, degree of conversion and microstructural changes were not directly characterized in this study; therefore, the proposed mechanisms remain hypotheses. The tested beverages were not chemically characterized, which limits the interpretation of the observed optical changes in relation to specific chromophores and tannins. Future studies should assess complex restoration geometries, local layer–surface interactions, validated cross-printer comparisons, and combined chemical and mechanical aging.

## 5. Conclusions

Within the limitations of this in vitro study, baseline optical properties varied by configuration. System S was lighter and had lower a^*^ and b^*^ values than system F, but these differences depended on build orientation and specimen thickness. System S demonstrated greater color stability at 0° during exposure to staining media, while system F showed greater color change, especially in red wine and at the 90°/1-mm configuration. A 0° build orientation generally favored color stability, while a 2-mm thickness most consistently limited changes in relative translucency. However, neither condition was uniformly protective across all systems and storage media. Clinically unacceptable color changes were more common than unacceptable translucency changes, and no 2-mm group exceeded the translucency acceptability threshold. The material system, fabrication workflow, restoration thickness, and local layer orientation should be jointly considered when planning 3D-printed restorations, especially for thin restorations in esthetically critical areas. These considerations are based on optical behavior observed under the tested laboratory conditions; their clinical applicability requires further validation. The findings apply to the complete restorative systems, as the effects of material composition could not be separated from those of the associated printing and post-polymerization workflows.

## Figures and Tables

**Table 1:** Material systems, reported composition, and fabrication workflows.

System	Restorative Resin-Based Composite	Reported Composition	Printer and Post-Processing Workflow
F	Permanent Crown Resin, shade A2 (Formlabs Inc., Somerville, MA, USA)	Bis-EMA, 50–75 wt%; silanized glass, 30–50 wt%; TPO, <2.5 wt%; pigments	Form 3; SLA; 50-µm layers. Form Wash in 99% isopropyl alcohol for 3 min; Form Cure for 20 min at 60 °C.
S	Saremco Print CROWNTEC, shade A2 (Saremco Dental AG, Rebstein, Switzerland)	Bis-EMA, 50–75 wt%; dental glass and silica fillers, 30–50 wt% (mean particle size, 0.7 µm); methyl benzoylformate and TPO, 1-5 wt% each; inhibitors; pigments	Asiga MAX UV; DLP; 50 µm layers. Manual alcohol/isopropyl alcohol cleaning; Otoflash G171 under nitrogen for 2 × 2000 flashes, with specimen rotation between cycles.

Bis-EMA, ethoxylated bisphenol A dimethacrylate; DLP, digital light processing; SLA, stereolithography; and TPO, diphenyl(2,4,6-trimethylbenzoyl) phosphine oxide. Composition was reported according to the manufacturers’ safety and technical data.

**Table 2:** Baseline CIELAB coordinates and three-way factorial ANOVA results.

**Baseline descriptive statistics**						
**Material system**	**Build** **orientation**	**Thickness (mm)**	***L*** ^*^		***a*** ^*^	***b*** ^*^
F	0°	1	86.60 ± 0.85		0.98 ± 0.12	31.83 ± 0.84
		2	81.63 ± 0.65		1.09 ± 0.17	30.80 ± 0.84
	90°	1	80.56 ± 3.13		1.75 ± 0.36	31.85 ± 0.61
		2	73.86 ± 1.69		1.90 ± 0.16	30.37 ± 0.76
S	0°	1	88.13 ± 0.26		−0.32 ± 0.07	29.45 ± 0.75
		2	84.20 ± 0.31		0.08 ± 0.11	28.73 ± 0.63
	90°	1	88.89 ± 0.60		0.35 ± 0.13	27.87 ± 1.06
		2	84.18 ± 0.50		0.39 ± 0.07	29.34 ± 0.66
**Three-way factorial ANOVA**						
**Outcome**	**Effect**	**df_1_**	**df_2_**	**F**	***p***	**Partial ηp^2^**
***L*** ^*^	Material system	1	216	993.097	<0.001	0.821
	Build orientation	1	216	328.132	<0.001	0.603
	Specimen thickness	1	216	790.687	<0.001	0.785
	Material system × Build orientation	1	216	405.345	<0.001	0.652
	Material system × Specimen thickness	1	216	17.564	<0.001	0.075
	Build orientation × Specimen thickness	1	216	12.099	<0.001	0.053
	Material system × Build orientation × Specimen thickness	1	216	1.693	0.195	0.008
***a*** ^*^	Material system	1	216	3213.231	<0.001	0.937
	Build orientation	1	216	771.777	<0.001	0.781
	Specimen thickness	1	216	58.262	<0.001	0.212
	Material system × Build orientation	1	216	41.868	<0.001	0.162
	Material system × Specimen thickness	1	216	3.040	0.083	0.014
	Build orientation × Specimen thickness	1	216	12.432	<0.001	0.054
	Material system × Build orientation × Specimen thickness	1	216	19.854	<0.001	0.084
***b*** ^*^	Material system	1	216	512.846	<0.001	0.704
	Build orientation	1	216	10.769	0.001	0.047
	Specimen thickness	1	216	17.534	<0.001	0.075
	Material system × Build orientation	1	216	1.777	0.184	0.008
	Material system × Specimen thickness	1	216	60.475	<0.001	0.219
	Build orientation × Specimen thickness	1	216	17.391	<0.001	0.075
	Material system × Build orientation × Specimen thickness	1	216	40.424	<0.001	0.158

Descriptive values are mean ± SD (n = 28 per material system × build orientation × thickness cell) and were obtained before immersion. F and S denote the two resin-system fabrication workflows described in Table 1. df_1_ and df_2_ are the numerator and denominator degrees of freedom, respectively. ηp^2^, partial eta squared.

**Table 3:** Color change (ΔE_00_) according to storage medium, material system, specimen thickness, build orientation, and immersion time.

Storage Medium	Thickness (mm)	Build Orientation	System F			System S		
			t_1_	t_7_	t_30_	t_1_	t_7_	t_30_
Distilled water	1	0°	0.36 ± 0.15 ^Aa^	0.45 ± 0.13 ^Aa^	0.95 ± 0.43 ^Aa^	0.73 ± 0.16 ^Ba^	1.05 ± 0.26 ^Ba^	1.23 ± 0.21 ^Aab^
		90°	0.76 ± 0.44 ^Ab^	0.85 ± 0.47 ^Aab^	1.89 ± 1.42 ^Ab^	0.93 ± 0.40 ^Aa^	1.68 ± 0.28 ^Bb^	1.83 ± 0.49 ^Aa^
	2	0°	0.43 ± 0.19 ^Aa^	0.41 ± 0.27 ^Aa^	1.29 ± 0.35 ^Aab^	0.31 ± 0.10 ^Ab^	0.59 ± 0.11 ^Aa^	0.66 ± 0.14 ^Aa^
		90°	0.71 ± 0.29 ^Aab^	1.17 ± 0.62 ^Ab^	1.83 ± 0.71 ^Aab^	0.56 ± 0.54 ^Ab^	1.81 ± 0.83 ^Bb^	1.62 ± 0.87 ^Aab^
Coffee	1	0°	2.26 ± 0.66 ^Ab^	5.10 ± 0.97 ^Ab^	9.14 ± 1.12 ^Ab^	0.35 ± 0.07 ^Ba^	0.60 ± 0.15 ^Ba^	1.53 ± 0.35 ^Bb^
		90°	4.85 ± 0.99 ^Ac^	9.47 ± 0.60 ^Ad^	14.92 ± 1.55 ^Ad^	3.67 ± 0.74 ^Bb^	7.13 ± 1.07 ^Bb^	11.14 ± 1.18 ^Bd^
	2	0°	1.36 ± 0.35 ^Aa^	3.70 ± 0.61 ^Aa^	6.43 ± 0.76 ^Aa^	0.31 ± 0.16 ^Ba^	0.51 ± 0.18 ^Ba^	0.61 ± 0.24 ^Ba^
		90°	4.24 ± 1.26 ^Ac^	8.32 ± 1.42 ^Ac^	13.13 ± 1.86 ^Ac^	3.54 ± 0.35 ^Ab^	6.34 ± 0.92 ^Bb^	9.61 ± 1.38 ^Bc^
Tea	1	0°	1.12 ± 0.13 ^Aa^	3.37 ± 0.81 ^Ab^	7.03 ± 0.48 ^Ab^	0.46 ± 0.11 ^Ba^	1.06 ± 0.40 ^Ba^	2.21 ± 0.33 ^Bb^
		90°	3.48 ± 0.53 ^Ab^	6.35 ± 1.63 ^Ac^	11.83 ± 1.20 ^Ad^	1.98 ± 0.75 ^Bb^	4.20 ± 0.97 ^Bb^	8.48 ± 2.21 ^Bd^
	2	0°	0.94 ± 0.33 ^Aa^	2.06 ± 0.35 ^Aa^	5.05 ± 0.77 ^Aa^	0.27 ± 0.12 ^Ba^	0.52 ± 0.18 ^Ba^	1.19 ± 0.20 ^Ba^
		90°	3.16 ± 0.89 ^Ab^	5.58 ± 0.96 ^Ac^	10.14 ± 1.15 ^Ac^	2.34 ± 0.56 ^Bb^	3.67 ± 0.76 ^Bb^	6.57 ± 1.20 ^Bc^
Red wine	1	0°	8.38 ± 0.92 ^Aa^	16.62 ± 3.40 ^Aa^	23.39 ± 4.85 ^Ab^	1.60 ± 0.27 ^Ba^	2.34 ± 0.38 ^Ba^	4.25 ± 0.56 ^Ba^
		90°	18.13 ± 6.16 ^Ac^	29.34 ± 8.56 ^Ac^	41.46 ± 9.48 ^Ad^	5.26 ± 0.68 ^Bc^	8.33 ± 1.17 ^Bc^	12.86 ± 1.45 ^Bd^
	2	0°	5.66 ± 1.24 ^Aa^	12.00 ± 2.33 ^Aa^	17.64 ± 2.40 ^Aa^	1.09 ± 0.40 ^Ba^	1.92 ± 1.18 ^Ba^	3.66 ± 1.44 ^Ba^
		90°	13.09 ± 3.92 ^Ab^	23.64 ± 7.62 ^Ab^	33.30 ± 6.80 ^Ac^	3.46 ± 0.41 ^Bb^	5.52 ± 0.82 ^Bb^	8.46 ± 0.80 ^Bc^

Values are mean ± SD. t_1_, t_7_, and t_30_ indicate change from baseline after 1, 7, and 30 days, respectively. Within each storage medium and time point, different uppercase superscripts indicate a significant difference between material systems for the same thickness–orientation combination; different lowercase superscripts indicate significant differences among thickness–orientation combinations within the same material system (Bonferroni-adjusted *p* < 0.05).

**Table 4:** Four-factor mixed ANOVA results for color change (ΔE_00_) within each storage medium.

Storage Medium	Effect	df_1_	df_2_	F	*p*	Partial ηp^2^
**Distilled water**	**Between-specimen effects**					
	Material system	1	48	2.469	0.123	0.049
	Specimen thickness	1	48	1.157	0.287	0.024
	Build orientation	1	48	34.572	<0.001	0.419
	Material system × Specimen thickness	1	48	4.189	0.046	0.080
	Material system × Build orientation	1	48	0.181	0.673	0.004
	Specimen thickness × Build orientation	1	48	0.476	0.494	0.010
	Material system × Specimen thickness × Build orientation	1	48	0.923	0.342	0.019
	**Within-specimen (time) effects**					
	Time	1.660	79.692	52.504	<0.001	0.522
	Time × Material system	1.660	79.692	10.734	<0.001	0.183
	Time × Specimen thickness	1.660	79.692	0.681	0.483	0.014
	Time × Build orientation	1.660	79.692	6.021	0.006	0.111
	Time × Material system × Specimen thickness	1.660	79.692	0.253	0.736	0.005
	Time × Material system × Build orientation	1.660	79.692	1.120	0.323	0.023
	Time × Specimen thickness × Build orientation	1.660	79.692	1.660	0.200	0.033
	Time × Material system × Specimen thickness × Build orientation	1.660	79.692	0.516	0.565	0.011
**Coffee**	**Between-specimen effects**					
	Material system	1	48	217.010	<0.001	0.819
	Specimen thickness	1	48	22.344	<0.001	0.318
	Build orientation	1	48	638.621	<0.001	0.930
	Material system × Specimen thickness	1	48	3.922	0.053	0.076
	Material system × Build orientation	1	48	17.175	<0.001	0.264
	Specimen thickness × Build orientation	1	48	0.001	0.980	0.000
	Material system × Specimen thickness × Build orientation	1	48	1.267	0.266	0.026
	**Within-specimen (time) effects**					
	Time	1.460	70.058	1339.294	<0.001	0.965
	Time × Material system	1.460	70.058	160.513	<0.001	0.770
	Time × Specimen thickness	1.460	70.058	18.205	<0.001	0.275
	Time × Build orientation	1.460	70.058	229.929	<0.001	0.827
	Time × Material system × Specimen thickness	1.460	70.058	0.322	0.657	0.007
	Time × Material system × Build orientation	1.460	70.058	16.220	<0.001	0.253
	Time × Specimen thickness × Build orientation	1.460	70.058	0.408	0.602	0.008
	Time × Material system × Specimen thickness × Build orientation	1.460	70.058	0.830	0.407	0.017
**Tea**	**Between-specimen effects**					
	Material system	1	48	154.376	<0.001	0.763
	Specimen thickness	1	48	21.247	<0.001	0.307
	Build orientation	1	48	377.532	<0.001	0.887
	Material system × Specimen thickness	1	48	1.228	0.273	0.025
	Material system × Build orientation	1	48	0.067	0.796	0.001
	Specimen thickness × Build orientation	1	48	0.027	0.870	0.001
	Material system × Specimen thickness × Build orientation	1	48	0.221	0.641	0.005
	**Within-specimen (time) effects**					
	Time	2	96	771.694	<0.001	0.941
	Time × Material system	2	96	73.113	<0.001	0.604
	Time × Specimen thickness	2	96	19.663	<0.001	0.291
	Time × Build orientation	2	96	91.970	<0.001	0.657
	Time × Material system × Specimen thickness	2	96	0.060	0.941	0.001
	Time × Material system × Build orientation	2	96	3.988	0.022	0.077
	Time × Specimen thickness × Build orientation	2	96	0.788	0.458	0.016
	Time × Material system × Specimen thickness × Build orientation	2	96	1.812	0.169	0.036
**Red wine**	**Between-specimen effects**					
	Material system	1	48	243.050	<0.001	0.835
	Specimen thickness	1	48	13.002	<0.001	0.213
	Build orientation	1	48	78.160	<0.001	0.620
	Material system × Specimen thickness	1	48	3.315	0.075	0.065
	Material system × Build orientation	1	48	15.376	<0.001	0.243
	Specimen thickness × Build orientation	1	48	1.270	0.265	0.026
	Material system × Specimen thickness × Build orientation	1	48	0.020	0.887	0.000
	**Within-specimen (time) effects**					
	Time	1.478	70.964	501.611	<0.001	0.913
	Time × Material system	1.478	70.964	179.449	<0.001	0.789
	Time × Specimen thickness	1.478	70.964	5.107	0.015	0.096
	Time × Build orientation	1.478	70.964	36.918	<0.001	0.435
	Time × Material system × Specimen thickness	1.478	70.964	0.770	0.431	0.016
	Time × Material system × Build orientation	1.478	70.964	5.478	0.012	0.102
	Time × Specimen thickness × Build orientation	1.478	70.964	0.620	0.494	0.013
	Time × Material system × Specimen thickness × Build orientation	1.478	70.964	0.487	0.560	0.010

Separate mixed ANOVAs were performed for each storage medium. Material system, specimen thickness, and build orientation were between-specimen factors; time was the within-specimen factor. df_1_ and df_2_ are the numerator and denominator degrees of freedom, respectively; non-integer degrees of freedom reflect sphericity correction. ηp^2^, partial eta squared.

**Table 5:** Relative translucency change (ΔRTP_00_) according to storage medium, material system, specimen thickness, build orientation, and immersion time.

Storage Medium	Thickness (mm)	Build Orientation	System F			System S		
			t_1_	t_7_	t_30_	t_1_	t_7_	t_30_
Distilled water	1	0°	0.46 ± 0.25 ^Aab^	0.28 ± 0.26 ^Aa^	1.08 ± 0.68 ^Ab^	1.30 ± 0.59 ^Bb^	0.49 ± 0.62 ^Aab^	0.57 ± 0.53 ^Ba^
		90°	0.55 ± 0.25 ^Aab^	0.86 ± 0.50 ^Ab^	0.68 ± 0.64 ^Aab^	0.43 ± 0.15 ^Aa^	0.96 ± 0.75 ^Ab^	1.09 ± 0.56 ^Ab^
	2	0°	0.21 ± 0.12 ^Aa^	0.23 ± 0.20 ^Aa^	0.30 ± 0.17 ^Aa^	0.46 ± 0.12 ^Aa^	0.09 ± 0.06 ^Aa^	0.11 ± 0.06 ^Aa^
		90°	0.61 ± 0.45 ^Ab^	0.32 ± 0.29 ^Aa^	0.27 ± 0.20 ^Aa^	0.23 ± 0.16 ^Ba^	0.52 ± 0.38 ^Aab^	0.48 ± 0.39 ^Aa^
Coffee	1	0°	0.28 ± 0.16 ^Aa^	1.28 ± 0.42 ^Ab^	2.54 ± 0.33 ^Ab^	0.83 ± 0.25 ^Bb^	0.41 ± 0.21 ^Ba^	0.98 ± 0.53 ^Ba^
		90°	0.76 ± 0.49 ^Ab^	1.14 ± 0.86 ^Aab^	3.58 ± 0.61 ^Ac^	0.92 ± 0.50 ^Ab^	2.17 ± 0.33 ^Bc^	3.42 ± 0.66 ^Ac^
	2	0°	0.21 ± 0.18 ^Aa^	0.71 ± 0.17 ^Aa^	1.26 ± 0.41 ^Aa^	0.30 ± 0.16 ^Aa^	0.33 ± 0.21 ^Aa^	0.53 ± 0.25 ^Ba^
		90°	0.56 ± 0.39 ^Ab^	1.10 ± 0.25 ^Aab^	1.38 ± 0.66 ^Aa^	0.32 ± 0.17 ^Aa^	0.97 ± 0.32 ^Ab^	1.44 ± 0.28 ^Ab^
Tea	1	0°	0.27 ± 0.21 ^Aa^	0.86 ± 0.38 ^Ab^	1.89 ± 0.56 ^Ac^	0.72 ± 0.61 ^Aa^	0.41 ± 0.24 ^Aa^	1.44 ± 0.64 ^Ac^
		90°	1.47 ± 1.01 ^Ab^	1.14 ± 1.26 ^Aab^	2.06 ± 0.65 ^Ac^	0.58 ± 0.43 ^Ba^	0.76 ± 0.55 ^Aa^	2.63 ± 0.59 ^Bd^
	2	0°	0.10 ± 0.06 ^Aa^	0.26 ± 0.17 ^Aa^	0.82 ± 0.20 ^Aa^	0.37 ± 0.20 ^Aa^	0.16 ± 0.10 ^Aa^	0.59 ± 0.22 ^Aa^
		90°	0.51 ± 0.38 ^Aa^	0.77 ± 0.34 ^Aab^	1.45 ± 0.57 ^Ab^	0.23 ± 0.16 ^Aa^	0.38 ± 0.13 ^Aa^	1.20 ± 0.39 ^Ab^
Red wine	1	0°	1.25 ± 0.68 ^Aa^	3.09 ± 0.79 ^Ab^	3.66 ± 0.96 ^Ab^	0.57 ± 0.42 ^Bab^	1.44 ± 0.30 ^Bb^	2.28 ± 0.82 ^Bb^
		90°	2.28 ± 1.14 ^Ab^	4.36 ± 0.69 ^Ac^	5.57 ± 0.85 ^Ac^	1.38 ± 0.64 ^Bc^	3.09 ± 0.81 ^Bc^	3.80 ± 0.94 ^Bc^
	2	0°	0.75 ± 0.31 ^Aa^	1.44 ± 0.42 ^Aa^	1.91 ± 0.31 ^Aa^	0.09 ± 0.06 ^Ba^	0.72 ± 0.10 ^Ba^	1.10 ± 0.11 ^Aa^
		90°	1.26 ± 0.27 ^Aa^	1.90 ± 0.82 ^Aa^	1.97 ± 1.10 ^Aa^	0.73 ± 0.09 ^Ab^	1.37 ± 0.24 ^Ab^	1.85 ± 0.31 ^Aab^

Values are mean ± SD. t_1_, t_7_, and t_30_ indicate change from baseline after 1, 7, and 30 days, respectively. Within each storage medium and time point, different uppercase superscripts indicate a significant difference between material systems for the same thickness–orientation combination; different lowercase superscripts indicate significant differences among thickness–orientation combinations within the same material system (Bonferroni-adjusted *p* < 0.05).

**Table 6:** Four-factor mixed ANOVA results for relative translucency change (ΔRTP_00_) within each storage medium.

Storage Medium	Effect	df_1_	df_2_	F	*p*	Partial ηp^2^
**Distilled water**	**Between-specimen effects**					
	Material system	1	48	0.914	0.344	0.019
	Specimen thickness	1	48	27.477	<0.001	0.364
	Build orientation	1	48	2.320	0.134	0.046
	Material system × Specimen thickness	1	48	1.063	0.308	0.022
	Material system × Build orientation	1	48	0.001	0.970	0.000
	Specimen thickness × Build orientation	1	48	0.478	0.493	0.010
	Material system × Specimen thickness × Build orientation	1	48	0.071	0.792	0.001
	**Within-specimen (time) effects**					
	Time	2	96	1.314	0.274	0.027
	Time × Material system	2	96	0.853	0.429	0.017
	Time × Specimen thickness	2	96	2.159	0.121	0.043
	Time × Build orientation	2	96	8.623	<0.001	0.152
	Time × Material system × Specimen thickness	2	96	1.757	0.178	0.035
	Time × Material system × Build orientation	2	96	15.344	<0.001	0.242
	Time × Specimen thickness × Build orientation	2	96	3.815	0.025	0.074
	Time × Material system × Specimen thickness × Build orientation	2	96	2.072	0.132	0.041
**Coffee**	**Between-specimen effects**					
	Material system	1	48	6.920	0.011	0.126
	Specimen thickness	1	48	123.415	<0.001	0.720
	Build orientation	1	48	95.926	<0.001	0.666
	Material system × Specimen thickness	1	48	0.347	0.559	0.007
	Material system × Build orientation	1	48	19.090	<0.001	0.285
	Specimen thickness × Build orientation	1	48	15.506	<0.001	0.244
	Material system × Specimen thickness × Build orientation	1	48	6.985	0.011	0.127
	**Within-specimen (time) effects**					
	Time	2	96	175.322	<0.001	0.785
	Time × Material system	2	96	12.837	<0.001	0.211
	Time × Specimen thickness	2	96	35.173	<0.001	0.423
	Time × Build orientation	2	96	18.182	<0.001	0.275
	Time × Material system × Specimen thickness	2	96	6.355	0.003	0.117
	Time × Material system × Build orientation	2	96	15.782	<0.001	0.247
	Time × Specimen thickness × Build orientation	2	96	8.204	<0.001	0.146
	Time × Material system × Specimen thickness × Build orientation	2	96	4.357	0.015	0.083
**Tea**	**Between-specimen effects**					
	Material system	1	48	4.462	0.040	0.085
	Specimen thickness	1	48	53.300	<0.001	0.526
	Build orientation	1	48	27.112	<0.001	0.361
	Material system × Specimen thickness	1	48	0.027	0.871	0.001
	Material system × Build orientation	1	48	1.194	0.280	0.024
	Specimen thickness × Build orientation	1	48	0.694	0.409	0.014
	Material system × Specimen thickness × Build orientation	1	48	0.343	0.561	0.007
	**Within-specimen (time) effects**					
	Time	1.725	82.788	71.069	<0.001	0.597
	Time × Material system	1.725	82.788	1.046	0.347	0.021
	Time × Specimen thickness	1.725	82.788	6.212	0.005	0.115
	Time × Build orientation	1.725	82.788	1.925	0.158	0.039
	Time × Material system × Specimen thickness	1.725	82.788	1.209	0.299	0.025
	Time × Material system × Build orientation	1.725	82.788	7.770	0.001	0.139
	Time × Specimen thickness × Build orientation	1.725	82.788	0.777	0.446	0.016
	Time × Material system × Specimen thickness × Build orientation	1.725	82.788	3.193	0.053	0.062
**Red wine**	**Between-specimen effects**					
	Material system	1	48	51.468	<0.001	0.517
	Specimen thickness	1	48	132.991	<0.001	0.735
	Build orientation	1	48	53.885	<0.001	0.529
	Material system × Specimen thickness	1	48	7.806	0.007	0.140
	Material system × Build orientation	1	48	0.252	0.618	0.005
	Specimen thickness × Build orientation	1	48	11.205	0.002	0.189
	Material system × Specimen thickness × Build orientation	1	48	0.609	0.439	0.013
	**Within-specimen (time) effects**					
	Time	2	96	156.416	<0.001	0.765
	Time × Material system	2	96	1.915	0.153	0.038
	Time × Specimen thickness	2	96	27.900	<0.001	0.368
	Time × Build orientation	2	96	1.384	0.255	0.028
	Time × Material system × Specimen thickness	2	96	2.777	0.067	0.055
	Time × Material system × Build orientation	2	96	0.365	0.695	0.008
	Time × Specimen thickness × Build orientation	2	96	2.964	0.056	0.058
	Time × Material system × Specimen thickness × Build orientation	2	96	1.253	0.290	0.025

Separate mixed ANOVAs were performed for each storage medium. Material system, specimen thickness, and build orientation were between-specimen factors; time was the within-specimen factor. df_1_ and df_2_ denote the numerator and denominator degrees of freedom, respectively. Non-integer degrees of freedom reflect Greenhouse–Geisser correction for violations of sphericity. η_p_^2^ denotes partial eta squared.

## Data Availability

The data supporting the findings of this study are available from the corresponding author upon reasonable request.
